# G4IPDB: A database for G-quadruplex structure forming nucleic acid interacting proteins

**DOI:** 10.1038/srep38144

**Published:** 2016-12-01

**Authors:** Subodh Kumar Mishra, Arpita Tawani, Amit Mishra, Amit Kumar

**Affiliations:** 1 Centre for Biosciences and Biomedical Engineering, Indian Institute of Technology Indore, Indore, Madhya Pradesh, 453552, India; 2 Cellular and Molecular Neurobiology Unit, Indian Institute of Technology, Jodhpur, Rajasthan, 342011, India

## Abstract

Nucleic acid G-quadruplex structure (G4) Interacting Proteins DataBase (G4IPDB) is an important database that contains detailed information about proteins interacting with nucleic acids that forms G-quadruplex structures. G4IPDB is the first database that provides comprehensive information about this interaction at a single platform. This database contains more than 200 entries with details of interaction such as interacting protein name and their synonyms, their UniProt-ID, source organism, target name and its sequences, ∆T_m_, binding/dissociation constants, protein gene name, protein FASTA sequence, interacting residue in protein, related PDB entries, interaction ID, graphical view, PMID, author’s name and techniques that were used to detect their interactions. G4IPDB also provides an efficient web-based “G-quadruplex predictor tool” that searches putative G-quadruplex forming sequences simultaneously in both sense and anti-sense strands of the query nucleotide sequence and provides the predicted G score. Studying the interaction between proteins and nucleic acids forming G-quadruplex structures could be of therapeutic significance for various diseases including cancer and neurological disease, therefore, having detail information about their interactions on a single platform would be helpful for the discovery and development of novel therapeutics. G4IPDB can be routinely updated (twice in year) and freely available on http://bsbe.iiti.ac.in/bsbe/ipdb/index.php.

Nucleic acids containing guanine rich sequences have potential to fold into inter- or intra- molecular secondary structure known as G-quadruplex structures^1^. These G-quadruplex structures are characterized by the presence of at least two stacks of four guanine nucleotides arranged in a coplanar manner. These stacked guanine nucleotides form G-tetrads that are stabilized by Hoogsteen system of hydrogen bonding as well as by the presence of monovalent cations that shields O6 carbonyl group of guanines^2^. G-quadruplex structures exhibit diverse topologies depending on the presence of monovalent cations (K^+^ or Na^+^), *syn* or *anti* conformation of glycosidic bond, number of strands involved in G-quadruplex formation (intermolecular, bimolecular or tetra molecular), comparative coordination link between the strands (parallel or antiparallel), number of stacking G-quartets and nucleotide sequences^3^,^4^. G-quadruplex forming DNA sequence are not evenly distributed throughout the genome rather they are profoundly located in the certain functional regions of chromosome such as telomeric regions, promoter region of various genes, intron and exon region of certain genes, etc^5^. G-quadruplex structures are known to be involved in replication, transcription, genetic recombination and other cellular activities^6^. It not only enhances the biological activities but also works as barricades to them, for example, certain endogenous G4 motifs formed within cells have ability to obstruct replication fork movement^7^. Apart from DNA, G-rich sequences of RNA also fold in to G-quadruplex structures. The first reported RNA G-quadruplex structure was a 19 nucleotide sequence at the 3′ terminus of 5 S rRNA of Escherichia coli^8^. Likely to DNA G-quadruplex motifs, the guanine rich RNAs are also involved in various biological activities and are known to be present in mRNA, long non-coding RNAs and in telomeric ends. In mRNA, G-quadruplex structures are mostly situated in the un-translated regions (UTRs), intronic regions, coding regions intronic regions and some in coding regions, and their presence strengthen their regulatory potentials^9^,^10^,^11^. However, the probability for the existence of RNA G-quadruplex structures is more than its DNA counterpart as RNA G-quadruplexes forms more thermodynamically stable, compact and less hydrated structures than DNA G-quadruplexes. Also, the presence of a 2′ hydroxyl group in the ribose sugars leads to more intra-molecular interactions and enhanced stability of RNA G-quadruplex structures. The discovery of disease - causing G-quadruplex DNA/RNA is yielding a wealth of new therapeutic targets, thereby, providing a new structure based tools for development of novel therapeutics.

In past, it was known that proteins bind to nucleic acids and play vital role in regulation of cell growth and development. Initially, proteins are known to bind to duplex DNA, however, there are proteins that binds to G-quadruplex DNA and/or RNA structures and play significant roles in various biological functions. The first reported G-quadruplex binding proteins were Telomeric DNA binding proteins that binds to the telomeric sequence and regulate the activity of telomerase enzyme^12^. This enzyme maintains the length of telomeres and counteracts its shortening during each cell division. Along with telomerases, Shelterin involves in group of six protein complex which plays crucial role for homoeostasis of telomeric length and prevent inappropriate activation of DNA damage response and repair^13^. It consists of TRF1 and TRF2, POT1 (protection of telomerase1), TPP1 (tripeptidyl peptidase 1), TIN2 (TERF1 (TRF1)-interacting nuclear factor 2) and RAP1 (Repressor/Activator Protein 1) proteins. Similarly, many proteins have also been reported that binds to other G-quadruplex motifs, for example, Nucleolin, that bind to NHE III region of C-MYC promoter forming G-quadruplex structure. Recently, TDP-43 have been discovered as G-quadruplex binding protein that interacts with GGGGCC rich transcript of C9ORF72 gene involved in ALS (Amyotrophic Lateral Sclerosis) disorder^14^.

Apart from DNA G-quadruplex binding proteins, RNA G-quadruplex binding proteins have also been reported. One of such proteins is fragile-X mental retardation protein (FMRP) that binds via its arginine–glycine–glycine (RGG) box to m-RNA forming G-quadruplex structure^15^. The interaction of RNA G- quadruplex structures with several ribosomal proteins has been revealed in 43 S pre-initiation complex that scans mRNA for start codon. The presence of G-quadruplex motif at 5′-UTR of this RNA prevents this recognition of the start codon and causes pathogenicity in the cell^16^. As these proteins have been found to be involved in several cellular processes, thus their study would lead to insights for the betterment of therapeutics development for various diseases. For example, the interaction of tumour suppressor proteins binding to nucleic acid sequence forming G-quadruplex structures could serve as a possible therapeutic target for cancer treatment^17^. In order to utilize this theme for the advancement of therapeutic development, it is requisite to understand the various parameters and conditions defining these interactions. Many researchers have explored a large number of such proteins and created a huge experimental dataset for their interactions with nucleic acids forming G-quadruplex structures. Assembling such huge information on a single platform would facilitate and expedite the strategies for drug discovery and development. Identification of new computation tools and construction of database containing information about G-quadruplex sequences, their structure and their interaction with various proteins would provide immense understanding for their formation, function and recognition. To the best of our knowledge, till date there is no such database available that is solely dedicated to the proteins interacting with nucleic acids forming G-quadruplex structures. Herein, we report the first database that provides detailed information for various proteins that binds to G-quadruplex structures forming DNA and/or RNA such as NCL^16^,^18^, RHAU^19^,^20^, BLM Helicase^21^, UP1^22^, TPP1^23^, IGF2^24^, FMRP^25^, SRSF1^16^, NOA1^26^, etc. These comprehensive details available on a single source would allow the database users to get all the relevant information in one click that ease drug discovery process in a rational manner.

## Results

### Browsing G4IPDB Database

**Figure 1:**
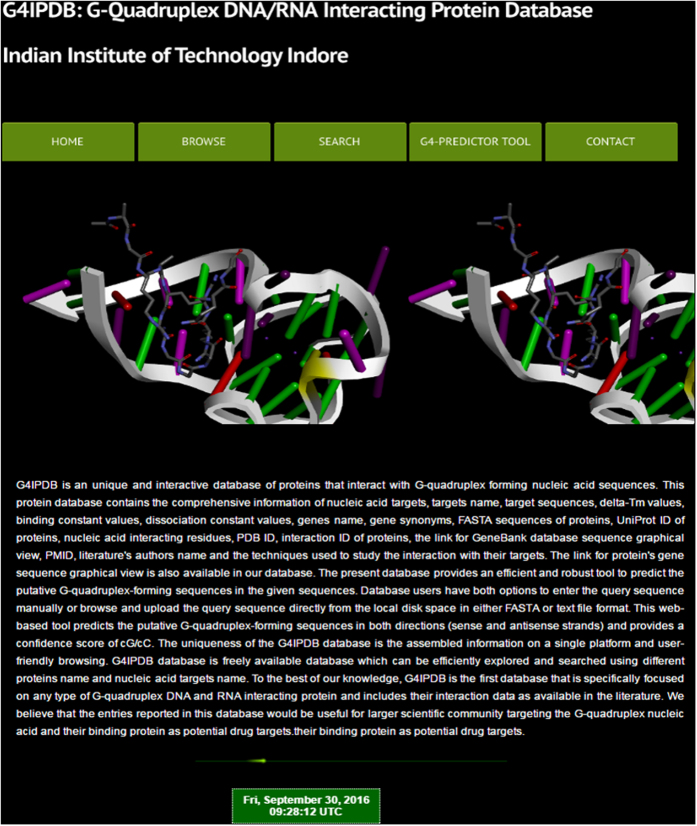
Home Page of G4IPDB. Depicting the browse, search, G4-predictor tool and contact options.

**Figure 2:**
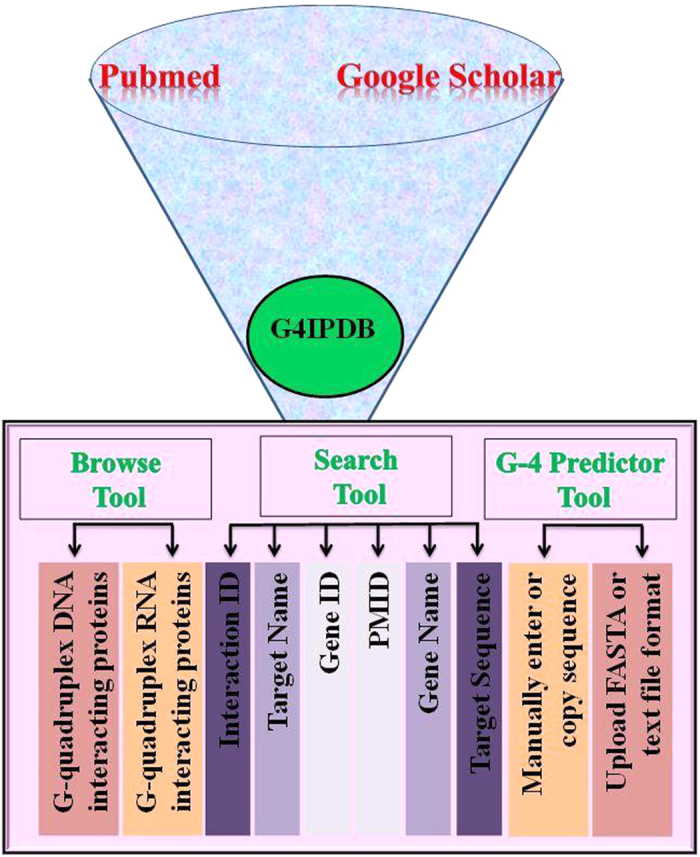
Schematic representation of the architecture of G4IPDB.

**Figure 3:**
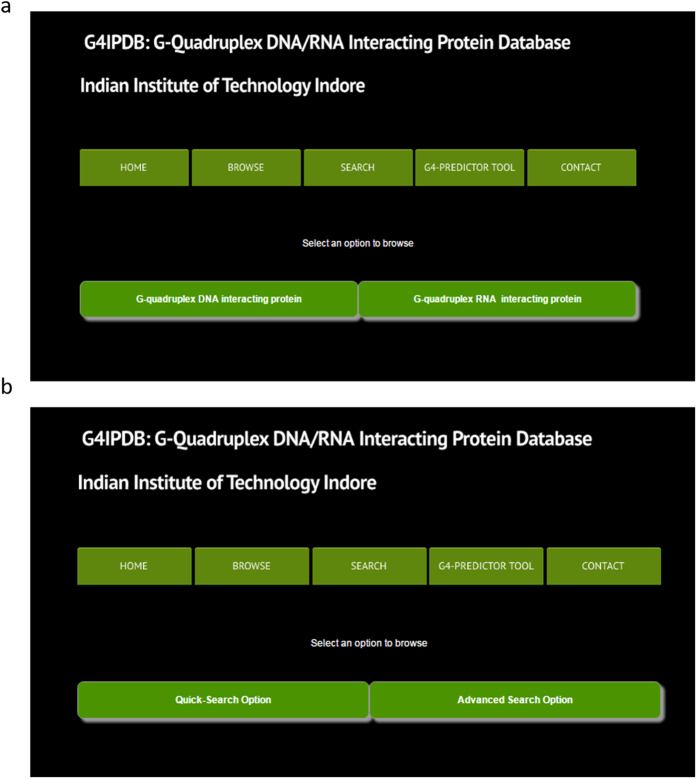
Browse and Search Options. Screenshots of (**a**) Browse option showing classified organization of G4IPDB and (**b**) Search options showing different searching criteria.

**Figure 4:**
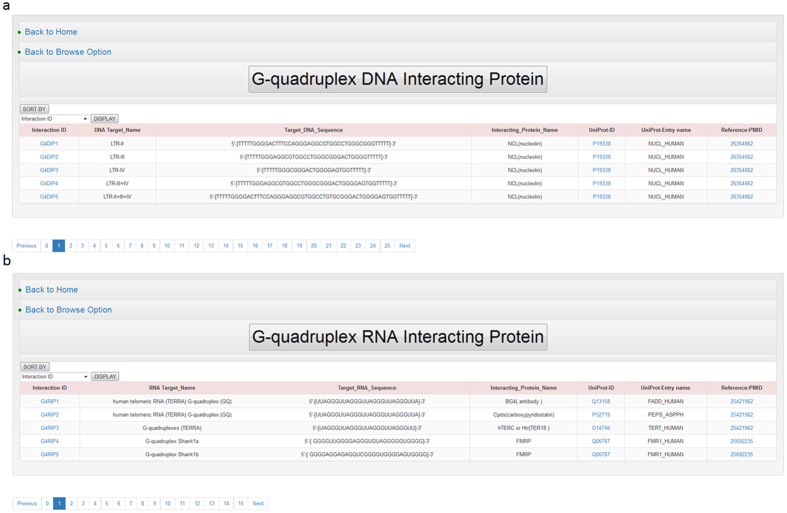
G-quadruplex DNA/RNA interacting proteins. Screenshots showing results of browsing (**a**) G-quadruplex DNA interacting protein and (**b**) G-quadruplex RNA interacting protein.

**Figure 5:**
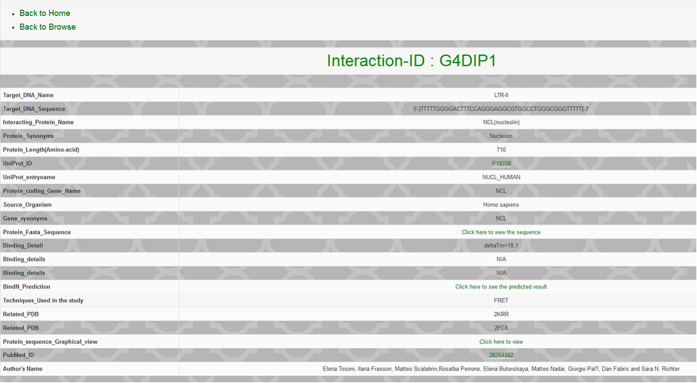
Various option available in Interaction ID. Screenshot showing various options of information about target nucleic acid and interacting proteins while browsing any Interaction ID present in the G4IPDB.

The Graphical User Interface (GUI) of G4IPDB database is available at the web URL http://bsbe.iiti.ac.in/bsbe/ipdb/index.php Fig. 1 depicts the screenshot of G4IPDB home page showing options for browse, search, G4 predictor tool and contact options. G4IPDB contains more than 200 entries that were broadly categorized into two browsing options (i) G-quadruplex DNA interacting protein and (ii) G-quadruplex RNA interacting protein. The architecture of G4IPDB is illustrated in Fig. 2 and screenshots of browse option page with the available browsing options is shown in Fig. 3a and b. These two categories contain entries for proteins specific to each type of G-quadruplex structures. These could be explored by browsing this tab that will open a new window containing list of G-quadruplex interacting proteins with their respective UniProt-ID, UniProt entry name, interaction ID, target DNA/RNA name, target DNA/RNA sequence (if available) and respective pubmed ID for reference from which the original data was collected (Fig. 4a and b). More details about their interaction could be explored by further browsing the hyperlinked interaction ID that will open a new table containing details of the interaction like dissociation and association constants (K_d_ and K_a,_ whichever is available in original reference), structural and sequence information about protein (PDB ID if available and their FASTA sequences, UniProt ID, UniProt Entry name, source organism, protein’s coding gene information (gene name and their synonyms), PMID, author’s name, techniques used to detect interactions etc. For example, if user will click on G4DIP1, which is the interaction ID for Nucleolin protein, a new window will open that list out various details about Nucleolin, its interacting nucleic acid structure and their interaction information (Fig. 5). The 3D structure of related proteins and their interactions with G-quadruplex DNA/RNA were hyperlinked as related PDB ID, if available in the literature. In order to assist the database users to retrieve the original source of experimental data, we have hyperlinked its reference PMID. For example, when browsing the interaction ID G4PIBD28, the resulting page will display the PMID as 25679041 that are hyperlinked with original PubMed link for its original research article which is “The maize (*Zea mays* L.) Nucleoside diphosphate kinase1 (zmNDPK1) gene encodes a human NM23-H2 homologue that binds and stabilizes G quadruplex DNA”. The Graphical User Interface (GUI) allows the users to perform text search, structure search for the G-quadruplex nucleic acids and protein structure as well as it also allows the downloading of entire database information.

### G4 sequence predictor tool

**Figure 6:**
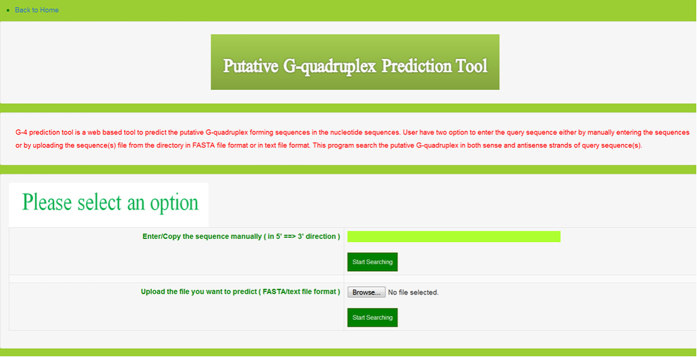
G4 predictor tool. Screenshots showing search methods in the G4 predictor tool.

**Figure 7:**
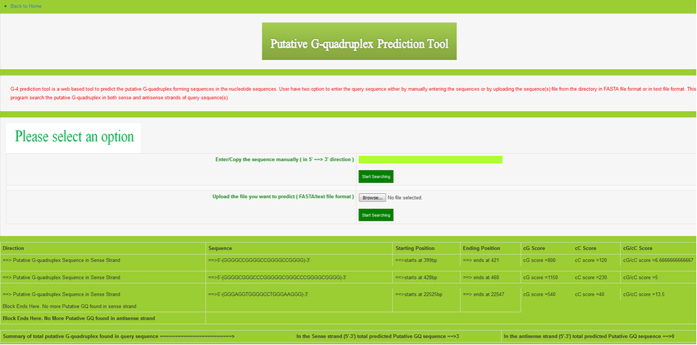
Results of G4 predictor tool. Screenshots of results showing various predicted putative G-quadruplex motif in the queried nucleotide sequences.

From last few decades, guanine rich sequences has gravitated the attention of scientific community because of their regulatory role in various biological processes and as a potent therapeutic target^27^,^28^. Therefore, building of efficient computational tool to mine the putative G-quadruplex forming sequences in the genome is highly important. G4IPDB provides a web-based tool that predicts the putative G-quadruplex forming sequences based on the previously described algorithm^29^. The screenshot of G4 predictor tool page shows different search options for predicting the putative G-quadruplex motif in given sequences provided by the database user (Fig. 6). G4-predictior tool is capable of predicting the putative G-quadruplex forming sequence simultaneously in both sense and antisense strands. It also shows the output of the start and end positions of each putative G-quadruplex forming motif and provides total number of putative G-quadruplex motif in the queried sequence. G4 predictor tool is extensively user friendly that allows user to either enter the sequence manually or browse the sequence containing file from the local disk. It is efficient to perform analysis of very large sequences on any genome size ranging from bacteria to mammalian genome. The prediction for putative G-quadruplex forming sequences is based on pattern matching of G_{Y1}_[X]_{Y2}_G_{Y1}_[X]_{Y2}_G_{Y1}_[X]_{Y2}_G_{Y1}_ motif. In this motif G represented the Guanine nucleotide and X represented any nucleotide including Adenine (A), Guanine (G), Cytosine(C), Thymine (T) and Uridine (U) nucleotide. The length of guanine tracts (Y1) varies from 2 to 7 in number and length of loop (Y2) varies with minimum of 1 and maximum of 7 nucleotides. The stability of predicted G-quadruplex structure will depend upon the number of guanine tracts, length of the internal loop as well as on the number of tandem repeats of the motif sequence. The default maximum length of putative G-quadruplex forming sequence is 49 bases. Figure 7 shows an example for the output of putative G-quadruplex forming sequence in the queried nucleotide sequence.

### G4 Prediction Score

The efficiently calculated cG and cC score has been validated as reliable and robust score for prediction of putative G-quadruplex forming sequence in given nucleotide sequences^30^. This score system lessen the false positive prediction and calculate the prediction score indubitably by considering few base pairs upstream and downstream of putative G4 motifs. The putative G-quadruplex motifs with higher cG/cG score have more probability to readily fold into G-quadruplex structure.

### Quick search option

In order to search database rapidly rather than constructing the proteins structure, we have provided Quick search option in the database. This search option facilitates the user to search database directly by protein or target name, target sequence, protein sequence, UniProt-ID, UniProt entry name, author’s name, technique used in the study, PMID etc. For example, if user gives FMRP as query string, this search option gives the output that has FMRP as interacting protein.

### Advanced search option

We have also provided an advanced search option to facilitate users to search database specifically and efficiently on the basis of users choice. In this search option we have provided 10 different types of search criteria such as G4IPDB interaction ID, DNA/RNA target name, DNA/RNA target sequence, Interacting protein name, UniProt-ID, UniProt entry name, protein coding gene name, gene synonyms, PMID and authors name. User can simultaneously select the range of search criteria and get the search result in more specific manner.

## Discussion

**Figure 8:**
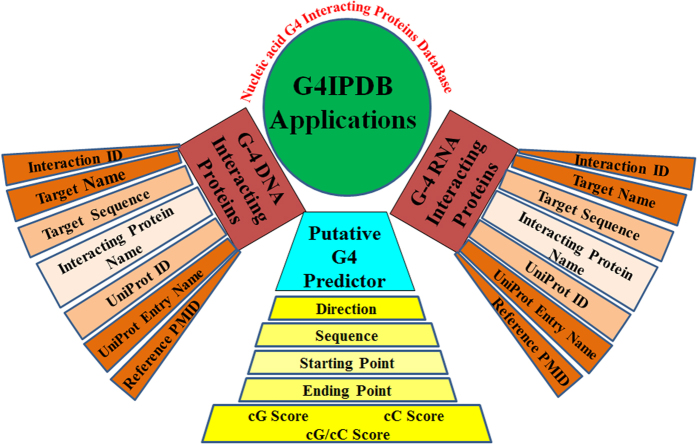
Schematic representation for the various applications of G4IPDB.

We have constructed G4IPDB database with an effort to assist the scientific community in further improving the efficiency of small molecule therapeutics for nucleic acid based diseases. The sequestration of proteins to G-quadruplex DNA/RNA motifs may lead to diseased conditions due to incorrect interactions. For instance, formation of G-quadruplex structure by the expanded repeat r(GGGGCC)_n_ in intron 1 of c9orf72 gene sequesters TDP-43 that leads to impairment of its function and causes ALS disorder^31^,^32^. However, certain G-quadruplex and protein interactions are also beneficial for cells, such as, interaction of promoter G-quadruplex motifs with various proteins and regulate their transcription process. For instance, the c-myc proto-oncogene is known to be overexpressed in more than 80% of tumors including colon cancer, breast cancer, etc^33^,^34^. Upstream region of its promoter controls its expression and contains G-quadruplex forming sequence. Nuclear protein Nucleolin stimulates G-quadruplex formation in the promoter region of c-myc gene and caused its transcriptional repression. Therefore, the understating of the interactions between these protein and G-quadruplex forming sequences would help us to design a better therapeutics for the diseases associated with G-quadruplex – protein interactions. Our database will provide a platform that contains information about these interactions and would be helpful for designing drugs and targeting diseases that involves G4-quadruplex-protein interactions. Figure 8 demonstrates an overview of various applications of G4IPDB. We have traversed more than 5000 peer-reviewed journals to gather the wide range of chemo-informatics data and compile them on a single platform. The G4IPDB is a collection of G-quadruplex DNA/RNA binding proteins with more than 200 entries. Users can browse this database on the basis of nucleic acid categories and have an ease to access several binding parameters such as their activity records, conventional pharmacodynamics and pharmacokinetic information includes binding constant, dissociation constant, T_m_ values, etc. The GUI of our database facilitates the user to search the database efficiently by protein name, UniProt ID, UniProt entry name, target name, target sequence, authors name and PMID. We believe that G4IPDB would stand as chemically oriented portal for the advancement of structure based drug design, virtual screening, molecular dynamic simulation and docking studies to develop the therapeutics for targeting nucleic acid based diseases. We are continuing in a process of growing this database with more entries for proteins. In future version of G4IPDB, we will include the tool for clustering analysis, QSAR statistical analysis and web-based docking tool to determine ligand target interactions.

## Methods

### Database overview

The database is built using XAMPP server (under the GPL license) which provides a user friendly integrated web development environment and supports MySQL, Apache, PHP at a single platform. Apache2 is used as web server platform and MySQL-RDMS (relational database management system) (5.6.24-MySQL Community Server) is used as database server for data storing; organization and query execution (see Supplementary Table S1–S5 for a complete description of MySQL database). G4IPDB web Server is running on the Dell Inc. (Model # PowerEdge R720xd) system which is equipped with Intel(R) Xeon(R) CPU E5-26650@2.40 GHz processor and 16 CPUsX2.399 GHz CPU cores. Website pages were built using PHP language on Net Beans IDE (8.1) platform. The G4IPDB site is best viewed by Google Chrome, Firefox, and Opera browser enabled with Java (version 1.6 or higher).

### Data collection

The information for G-quadruplex DNA/RNA- protein interaction were fetched from reported literature searched in PubMed and Google Scholar using various keywords such as ‘G-quadruplexes protein interaction’, ‘G-quadruplex DNA binding protein’, ‘Quadruplex DNA interaction with protein’, ‘G-quadruplex RNA binding proteins’ and ‘Quadruplex RNA interacting proteins’. The details of interacting proteins, UniProt-ID, UniProt entry name, protein coding gene name and gene synonyms, source organism, their target nucleotide sequences, association constant (K_a_), dissociation constant(K_d_), change in the melting temperature upon interaction of protein to their target nucleotide sequences (∆T_m_), related PDB ID, technique used to detect interaction, PMID, author’s name and other available information were manually mined from available literature (see Supplementary File for definition of descriptors). The entry for which PDB ID was not available, interacting residues in the binding protein was predicted by the web based tool BindN. The FASTA sequences of proteins were used for the prediction of interactive residue. For DNA binding proteins, prediction accuracy estimated from cross validation is about 70% with equal sensitivity and specificity. At the same time, for RNA residues the prediction accuracy estimated from cross validation is 68%. The external link for gene ID and NCBI sequences were manually collected and hyperlinked with the associated entries.

### G4-Predictor Tool

G4 Predictor tool was written in the PHP language and designed to search putative G-quadruplex forming motif in the both sense and anti-sense strands simultaneously. This is based on the searching of regular expression pattern and found non-overlapping putative G-quadruplex motif in DNA and RNA sequence. We have used the following regular expression for predicting the putative G-quadruplex forming sequences:


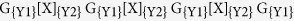


Where X is representing any nucleotide including A, G, C, T, and U and value of Y1 is varies from any number between 2 to 7 for variable length of guanine tract and value of Y2 is varies from any numbers between 1 to 7 for variable length of loop.

### G4 Prediction Score

In combination of the mining and prediction of putative G-quadruplex motif, G4 predictor tool also calculates the ‘cG’, ‘cC’, and ‘cG/cC’ scores based on the previous study of new scoring function for G-quadruplex motif^30^. Briefly, the cG score calculation is based on the following equation and applied for each predicted substring (s) that has the length of n:


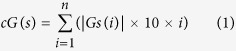


In this equation (1) a value of 10 is assigned to the each G, a value of 20 assigned for each paired GG and a value of 30 assigned for each triplet GGG and so on. The cC score calculation is also based on the similar equation only difference is that the cytosine nucleotide is used in place of guanine nucleotide. The cG/cC score is based on the ratio of both cG and cC scores.

## Additional Information

**How to cite this article**: Mishra, S. K. *et al*. G4IPDB: A database for G-quadruplex structure forming nucleic acid interacting proteins. *Sci. Rep.* **6**, 38144; doi: 10.1038/srep38144 (2016).

**Publisher's note:** Springer Nature remains neutral with regard to jurisdictional claims in published maps and institutional affiliations.

## Supplementary Material

Supplementary Information
