# Supplementary material for: G4IPDB: A database for G-quadruplex structure forming nucleic acid interacting proteins
Source: Sci Rep. 2016 Dec 1;6:38144. doi: 10.1038/srep38144 (PMC5131279; doi:10.1038/srep38144)
Supplement: Supplementary Information [file srep38144-s1.pdf]

G4IPDB: A database for G-quadruplex structure forming nucleic acid interacting proteins

Subodh Kumar Mishra[1], Arpita Tawani[1], Amit Mishra[2] & Amit Kumar[1,]*

[1]Centre for Biosciences and Biomedical Engineering, Indian Institute of Technology Indore, Indore, Madhya Pradesh, 453552, India
[2]Cellular and Molecular Neurobiology Unit, Indian Institute of Technology Jodhpur, Rajasthan, 342011, India

* To whom correspondence should be addressed. Dr. Amit Kumar, Tel:+91-731-2438771; Fax:+91-731-2364182; Email: amitk@iiti.ac.in

G4IPDB database description

MySQL RDBMS (relational database management system) is used for storing data. The main tables are as follows:

1) allip2 (stores information about all type of target nucleic acid)
2) g4dip (stores information about target G quadruplex DNA)
3) g4rip (stores information about target G quadruplex RNA)
4) g4dnabrowse (stores interaction information about Target G quadruplex DNA and proteins)
5) g4rnabrowse (stores interaction information about Target G quadruplex RNA and proteins)

Note: The following notations are used:

PRI: primary key, generally auto-generated by the database
UNI: this field will take only unique values
NULL: used to represent no value
varchar: MySQL data type, used for storing a string of limited length efficiently
int: MySQL data type, used to represent an integer

The Description of tables' fields is given below:

Supplementary Table S1: Allip2

Field	Type	Null	Collation	Default
id	varchar(500)	No	latin1_swedish_ci	None
targetname	varchar(500)	No	latin1_swedish_ci	--
targetseq	varchar(500)	Yes	latin1_swedish_ci	--
interactingproteinname	varchar(500)	No	latin1_swedish_ci	N/A

Synonyms	varchar(500)	No	latin1_swedish_ci	N/A
length	varchar(500)	No	latin1_swedish_ci	N/A
Uniprotcode	int(500)	Yes	latin1_swedish_ci	N/A
Uniprotname	varchar(500)	No	latin1_swedish_ci	N/A
genename	varchar(500)	No	latin1_swedish_ci	N/A
organism	varchar(500)	No	latin1_swedish_ci	N/A
Gene_synonyms	varchar(500)	Yes	latin1_swedish_ci	N/A
uniprotfastalink	varchar(500)	No	latin1_swedish_ci	N/A
bind1	varchar(500)	No	latin1_swedish_ci	
bind2	varchar(500)	No	latin1_swedish_ci	
bind3	varchar(500)	Yes	latin1_swedish_ci	N/A
bindn	varchar(500)	No	latin1_swedish_ci	N/A
technique	varchar(500)	No	latin1_swedish_ci	--
pdb1	varchar(500)	No	latin1_swedish_ci	--
pdb2	varchar(500)	No	latin1_swedish_ci	--
graph	varchar(500)	No	latin1_swedish_ci	--
pmid	varchar(500)	No	latin1_swedish_ci	--

authorname	varchar(500)	No	latin1_swedish_ci	--

Supplementary Table S2: g4dip

Field	Type	Null	Collation	Default
id	varchar(500)	No	latin1_swedish_ci	None
targetname	varchar(500)	No	latin1_swedish_ci	--
targetseq	varchar(500)	Yes	latin1_swedish_ci	--
interactingproteinname	varchar(500)	No	latin1_swedish_ci	N/A
Synonyms	varchar(500)	No	latin1_swedish_ci	N/A
length	varchar(500)	No	latin1_swedish_ci	N/A
Uniprotcode	int(500)	Yes	latin1_swedish_ci	N/A
Uniprotname	varchar(500)	No	latin1_swedish_ci	N/A
genename	varchar(500)	No	latin1_swedish_ci	N/A
organism	varchar(500)	No	latin1_swedish_ci	N/A
Gene_synonyms	varchar(500)	Yes	latin1_swedish_ci	N/A
uniprotfastalink	varchar(500)	No	latin1_swedish_ci	N/A
bind1	varchar(500)	No	latin1_swedish_ci	
bind2	varchar(500)	No	latin1_swedish_ci	
bind3	varchar(500)	Yes	latin1_swedish_ci	N/A

bindn	varchar(500)	No	latin1_swedish_ci	N/A
technique	varchar(500)	No	latin1_swedish_ci	--
pdb1	varchar(500)	No	latin1_swedish_ci	--
pdb2	varchar(500)	No	latin1_swedish_ci	--
graph	varchar(500)	No	latin1_swedish_ci	--
pmid	varchar(500)	No	latin1_swedish_ci	--
authorname	varchar(500)	No	latin1_swedish_ci	--

Supplementary Table S3: g4rip

Field	Type	Null	Collation	Default
id	varchar(500)	No	latin1_swedish_ci	None
targetname	varchar(500)	No	latin1_swedish_ci	--
targetseq	varchar(500)	Yes	latin1_swedish_ci	--
interactingproteinname	varchar(500)	No	latin1_swedish_ci	N/A
Synonyms	varchar(500)	No	latin1_swedish_ci	N/A
length	varchar(500)	No	latin1_swedish_ci	N/A
Uniprotcode	int(500)	Yes	latin1_swedish_ci	N/A
Uniprotname	varchar(500)	No	latin1_swedish_ci	N/A
genename	varchar(500)	No	latin1_swedish_ci	N/A

organism	varchar(500)	No	latin1_swedish_ci	N/A
Gene_synonyms	varchar(500)	Yes	latin1_swedish_ci	N/A
uniprotfastalink	varchar(500)	No	latin1_swedish_ci	N/A
bind1	varchar(500)	No	latin1_swedish_ci	
bind2	varchar(500)	No	latin1_swedish_ci	
bind3	varchar(500)	Yes	latin1_swedish_ci	N/A
bindn	varchar(500)	No	latin1_swedish_ci	N/A
technique	varchar(500)	No	latin1_swedish_ci	--
pdb1	varchar(500)	No	latin1_swedish_ci	--
pdb2	varchar(500)	No	latin1_swedish_ci	--
graph	varchar(500)	No	latin1_swedish_ci	--
pmid	varchar(500)	No	latin1_swedish_ci	--
authorname	varchar(500)	No	latin1_swedish_ci	--

Supplementary Table S4: g4dnabrowse

Field	Type	Null	Collation	Default
interaction_id	varchar(500)	No	latin1_swedish_ci	None
g4dna_name	varchar(500)	No	latin1_swedish_ci	--
Interacting_protein	varchar(500)	Yes	latin1_swedish_ci	--
target_dna_seq	varchar(500)	No	latin1_swedish_ci	N/A
Refference	int(500)	No	latin1_swedish_ci	N/A

UniProtcode	varchar(500)	No	latin1_swedish_ci	N/A
uniprot-entryname	varchar(500)	No	latin1_swedish_ci	N/A

Supplementary Table S5: g4rnabrowse

Field	Type	Null	Collation	Default
interaction_id	varchar(500)	No	latin1_swedish_ci	None
g4dna_name	varchar(500)	No	latin1_swedish_ci	--
Interacting_protein	varchar(500)	Yes	latin1_swedish_ci	--
target_dna_seq	varchar(500)	No	latin1_swedish_ci	N/A
Refference	int(500)	No	latin1_swedish_ci	N/A
UniProtcode	varchar(500)	No	latin1_swedish_ci	N/A
uniprot-entryname	varchar(500)	No	latin1_swedish_ci	N/A

Descriptors definition

1. **Protein FASTA sequense**

FASTA sequence file format starts with greater-than (">") symbol and have one line description about sequence (less than 80 character set)and followed by the sequence data. For instance an example of FASTA sequence given below:

>gi|649115499|gb|AIC53396.1| C9orf72, partial [synthetic construct]

MSTLCPPPSPAVAKTEIALSGKSPLLAATFAYWDNILGPRVRHIWAPKTEQVLLSDGEITFLANHTLNGEILR

NAESGAIDVKFFVLSEKGVIIVSLIFDGNWNGDRSTYGLSIILPQTELSFYLPLHRVCVDRLTHIIRKGRIWM

HKERQENVQKIILEGTERMEDQGQSIIPMLTGEVIPVMELLSSMKSHSVPEEIDIADTVLNDDDIGDSCHEGF

LLNAISSHLQTCGCSVVVGSSAEKVNKIVRTLCLFLTPAERKCSRLCEAESSFKYESGLFVQGLLKDSTGSFV

LPFRQVMYAPYPTTHIDVDVNTVKQMPPCHEHIYNQRRYMRSELTAFWRATSEEDMAQDTIIYTDESFTPDLN

IFQDVLHRDTLVKAFLDQVFQLKPGLSLRSTFLAQFLLVLHRKALTLIKYIEDDTQKGKKPFKSLRNLKIDLD

LTAEGDLNIIMALAEKIKPGLHSFIFGRPFYTSVQERDVLMTF

Space and blank lines are not allowed in the sequence data and sequence data must be represented in the IUB/IUPAC amino acid and nucleic acid codes. A single hyphen or dash can be used to represent a gap of indeterminate length; and in amino acid sequences, U and * are acceptable letters (see below). The list of accepted code are as in below table.

Supplementary Table S6: one letter Code for the amino acid

Code: Discription	Code: Discription
A: alanine	R: arginine
P: proline	D: aspartate
B: aspartate/asparagine	S: serine
Q: glutamine	E: glutamate
C: cystine	T: threonine
V: valine	F: phenylalanine
H: histidine	U: selenocysteine
W: tryptophan	G: glycine
I: isoleucine	X: any
Y: tyrosine	N: asparagine
L: leucine	Z: glutamate/glutamine
K: lysine	*: translation stop
M: methionine	-: gap of indeterminate length

2. **Gene ID**

   This is the unique identifier assign to the each gene present in the Gene database. User can directly access the entries in the Gene database by entering the respective Gene ID. This identifier ease the access various gene detail such as gene official symbols, primary source, organism, lineage, gene summary, and gene genomic context etc.

3. **PDB ID**

   PDB (Protein Data Bank) , is a structural database and have the information about the 3D structure of proteins, nucleic acid, and their complex. A 4 letter alphanumeric unique code attributed to each entry in the PDB database. User can directly search the protein or nucleic acid structure by directly using the respective PDB ID.

4. **UniProtID**

   UniProt is freely available comprehensive database of proteins containing information about there their sequence and biological function extracted from the available research data. This database provides a unique ID to each entry in the database which contains alphanumeric characters. For example P07271, P54132, O94761, etc.
